# Beeswax cleaning by solvent extraction of pesticides

**DOI:** 10.1016/j.mex.2019.04.022

**Published:** 2019-04-24

**Authors:** Pau Calatayud-Vernich, Dennis VanEngelsdorp, Yolanda Picó

**Affiliations:** a Environmental and Food Safety Research Group (SAMA-UV), Research Center on Desertification (CIDE, CSIC-University of Valencia-Generalitat Valenciana), Moncada-Naquera Road Km 4.5, 46113 Moncada, Valencia, Spain; b Department of Entomology, Plant Science Building, University of Maryland, MD 20742, United States; c CIBER of Epidemiology and Public Health (CIBERESP), Av. Monforte de Lemos, 3-5. Pabellón 11, 28029 Madrid, Spain

**Keywords:** Beeswax cleaning by solvent extraction, Beeswax, Solvent extraction, Pesticides removal, Hexane, *N*, *N*-Dimethylformamide (DMF)

## Abstract

We set out to test if the methodology used to clean sheep wool wax (Lanolin) from pesticides could be used to clean beeswax as well. We first made an aggregate sample of brood comb wax from three different US beekeepers. Sub-samples of these aggregate wax samples were analyzed for pesticide contamination. The remaining wax, was then dissolved into hexane solution and run through four *N*, *N*-Dimethylformamide (DMF) washes. During these extractions, the pesticides partitioned into the DMF, and so were removed from the beeswax. Following the solvent extractions, the beeswax was tested again for pesticides. An average of 95% of the pesticide contamination was removed by the chemical wash procedure.

• Beeswax is the beekeeping matrix with the highest pesticide content.
• This study developed methodology for solvent-based removal of pesticides from beeswax (>95%).
• Of 24 pesticides detected in beeswax samples before to the solvent extraction, only 3 pesticides were detected after the extraction with DMF.

## Graphical abstract

**Figure:**
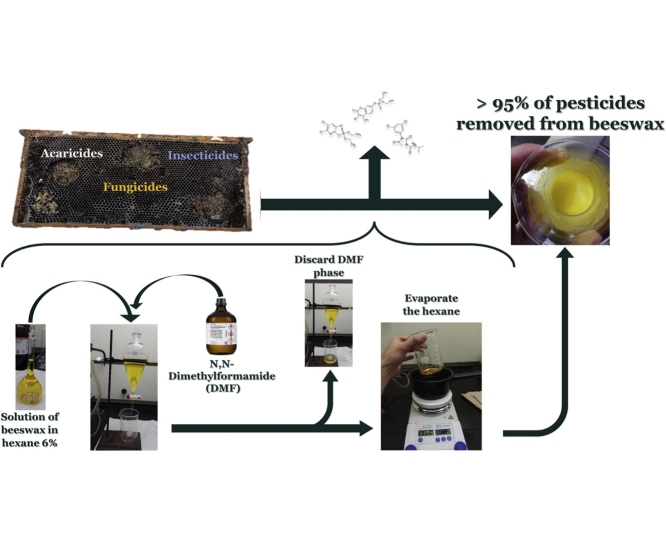

**Specifications Table**

**Table:** 

Subject Area:	*Chemistry*
More specific subject area:	*Analytical chemistry*
Method name:	*Beeswax cleaning by solvent extraction*
Name and reference of original method:	*Jones, F. (1997). The removal of pesticide residues from wool wax by solvent extraction. J. Am. Oil Chem. Soc. 74, 1241-1245.*
Resource availability:	*Basic laboratory equipment like a fume hood, spatules, funnels, paper filters, pippetes, etc ...*

## Method details

### Material

• Beeswax from old brood combs.
• Hexane and *N*, *N*-Dimethylformamide (DMF).
• Beakers 50–500 mL.
• Analytical scale.
• Separating funnel (1 L).
• Flasks 250 mL.

Note: Availability of Standard laboratory equipment is assumed.

### Purification of the beeswax samples

**Fig. 1:**
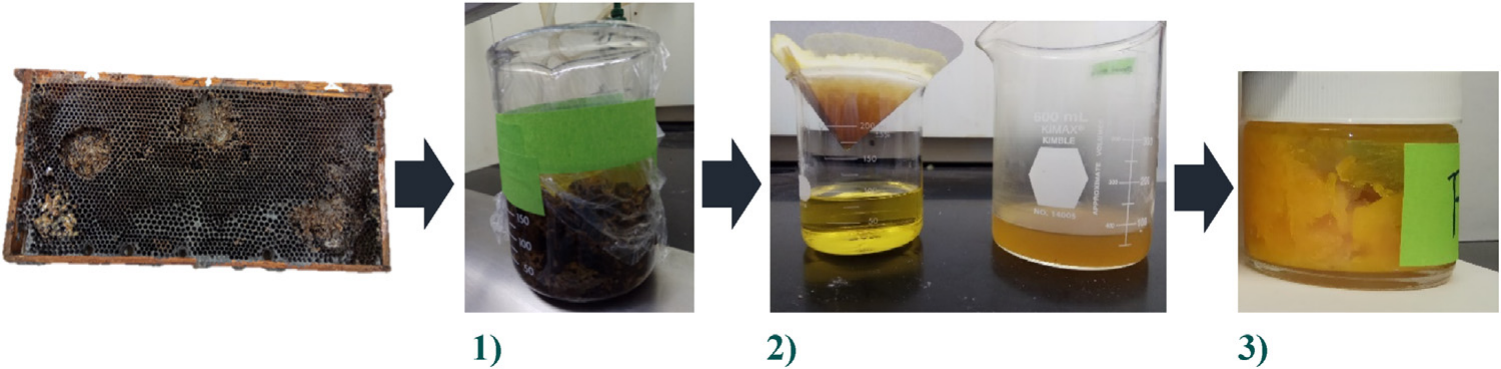
Process to obtain the three batches of purified beeswax that were subjected to the solvent extraction of the pesticides. Wax was removed from old dark brood comb and dissolved into a hexane solution (1), which was then filtered (2), and had the hexane removed by evaporation under a stream of air. The resulting “pure” wax (3) was used to test for pesticides, and the pesticide wash study.

Prior to solvent removal of the pesticides from beeswax, three homogenous pools of beeswax from three different combs were prepared. When the brood comb beeswax source is old, as was the case for the combs collected from three different commercial beekeepers in this study, it is contaminated with many impurities such as bee silk, produced by pupating bees, propolis, pollen, and larvae excrement. These impurities are difficult to remove, and so, to obtain “clean beeswax” we used the follow procedure (Fig. 1). While beekeepers typically boil old combs (called slumgum), this process removes little wax, as the wax is absorbed by the bee silk cocoons that line the brood comb cells. Beekeepers eager to remove this wax, often use steam and pressure to separate impurities from the wax [1]. The separation of the beeswax from comb impurities using steam and pressure were not practical in our case, and so, we used the following chemical solvent method (Fig. 1):

1 Wax contained in the combs was obtained by dissolving the brood comb with the hexane solvent.
2 After the wax was dissolved in hexane, the solution was filtered to eliminate impurities.
3 Hexane was evaporated to obtain the three batches of beeswax. By weight, between a 15–19% of the old brood comb was recoverable beeswax. Of the 150 g of comb was collected per operation, 23 g of “pure” beeswax was recovered and used for further study.

### Solvent extraction of pesticides from beeswax samples

**Fig. 2:**
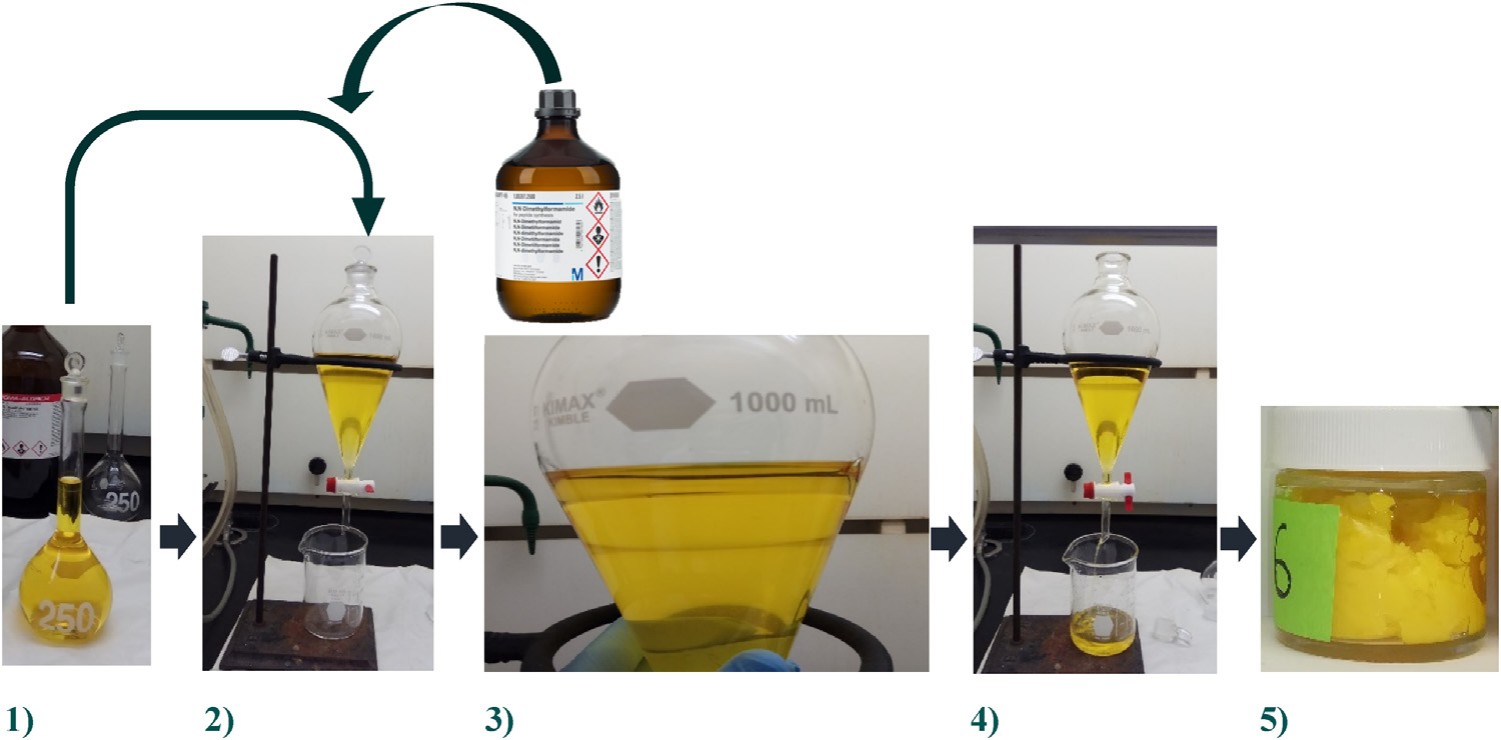
Solvent extraction of the pesticides from beeswax. The wax-hexane solution (1) is placed into a separating funnel which also contains DMF (2). After vigorous agitation, the solution is let rest to separate the two phases (3), the DMF is then drained (4). The hexane is removed from the resulting solution by evaporation under an air steam resulting in a semi-solid beeswax (5).
4) Drain the DMF phase. Repeat the extraction process four times.
5) Keep the hexane-wax phase and evaporate hexane by evaporation under an air steam.

Contaminant removal methods have successfully been used with lanolin (wool wax) [2], and for the current project, these methods were adapted and tested for their ability to remove pesticide contaminants of beeswax. The following procedure was repeated for each batch of beeswax removed from the three brood combs:

1) Prepare a solution of “pure” wax (see procedure above) in hexane 6% (250 mL, wt/vol).
2) Mix and agitate the 250 ml of wax solution added to 250 mL of DMF (extractant solvent) in a separating funnel (1 L).
3) Let the DMF and the hexane solution separate into two phases by letting the solution rest until the two physical layers are observed (hexane-wax solution, upper phase; DMF, lower phase) (Fig. 2).

### Method validation

Beeswax samples obtained before, and after the solvent extractions were sent to the USDA-AMS National Science Laboratory in Gastonia NC for multi-pesticide residue analysis to evaluate the effectiveness of the cleaning method. Wax was analyzed for 199 pesticides and associated degradates as described in Mullin et al. [3]. As can be observed in Table 1, Table 2, Table 3, the solvent cleaning tested here is an effective method to decontaminate beeswax from pesticides. Pesticide incidence was reduced significantly in the three beeswax samples. Of 24 different pesticides detected before to the solvent extraction, only 3 pesticides were found after the decontamination procedure proposed was applied.

**Table 1:** Summary of pesticide detections in wax comb 1 before and after the solvent extraction of pesticides.

WAX COMB 1				
Pesticides and degradates	LOD (ng·g^−1^)	Detections before solvent extraction (ng·g^−1^)	Detections after solvent extraction (ng·g^−1^)	Removal (%)
2,4 Dimethylphenyl formamide (DMPF)	1.5	111	0	100
Azoxystrobin	1	2	0	100
Chlorthal-dimethyl (DCPA)	2	Trace	0	–
Coumaphos	4	1420	Trace	–
Coumaphos oxon	0.5	69	0	100
DEET	3	23	7	70
Diphenylamine	2	9	0	100
Fenpyroximate	3	264	0	100
Fluvalinate	25	1180	0	100
Iprodione	100	137	0	100
Metolachlor	25	Trace	0	–
Thymol	2	2750	0	100
Trifloxystrobin	1	1	0	100
			**Removal Average**	97

**Table 2:** Summary of pesticide detections in wax comb 2 before and after the solvent extraction of pesticides.

WAX COMB 2				
Pesticides and degradates	LOD (ng·g^−1^)	Detections before solvent extraction (ng·g^−1^)	Detections after solvent extraction (ng·g^−1^)	Removal (%)
2,4 Dimethylphenyl formamide (DMPF)	1.5	41	0	100
Azoxystrobin	1	8	0	100
Boscalid	5	25	0	100
Carbendazim	2	Trace	0	–
Chlorpyrifos	5	Trace	0	–
Chlorthal-dimethyl (DCPA)	2	3	0	100
Coumaphos	4	5270	Trace	–
Coumaphos oxon	0.5	157	0	100
Cyprodinil	2	121	0	100
DEET	3	5	5	0
Diphenylamine	2	7	0	100
Fenpyroximate	3	1330	0	100
Fluvalinate	25	2900	0	100
Iprodione	100	3330	0	100
Methoxyfenozide	1	4	0	100
Myclobutanil	7	41	0	100
Propiconazole	2	7	6	14
Pyraclostrobin	2	75	0	100
Pyridaben	2	9	0	100
Pyrimethanil	5	41	0	100
Thymol	2	12500	0	100
Trifloxystrobin	1	6	0	100
			**Removal average**	90

**Table 3:** Summary of pesticide detections in wax comb 3 before and after the solvent extraction of pesticides.

WAX COMB 3				
Pesticides and degradates	LOD (ng·g^−1^)	Detections before solvent extraction (ng·g^−1^)	Detections after solvent extraction (ng·g^−1^)	Removal (%)
2,4 Dimethylphenyl formamide (DMPF)	1.5	300	0	100
Boscalid	5	9	0	100
Coumaphos	4	1160	0	100
Coumaphos oxon	0.5	24	0	100
Cyprodinil	2	99	0	100
Fenpyroximate	3	610	0	100
Fluopyram	1	3	0	100
Fluvalinate	25	562	0	100
Iprodione	100	419	0	100
Methoxyfenozide	1	16	0	100
Myclobutanil	7	30	0	100
Penthiopyrad	1	57	0	100
Propiconazole	2	39	0	100
Pyraclostrobin	2	5	0	100
Thymol	2	766	0	100
Trifloxystrobin	1	6	0	100
			**Removal average**	100

## Additional Information

Given the use of pesticides in beekeeping and plant protection, hive products exposure to pesticides is unavoidable. Acaricides, fungicides and insecticides have been found in beeswax from Europe [4,5] and America [6]. Wax is the most contaminated hive matrix, and its nature based on lipids and hydrocarbons, is in part, responsible of its high pesticide content. The most common wax contaminants are lipophilic, and do not degrade during the wax recycling. Moreover, some of the pesticides found in beeswax have not been used in years, suggesting its bioaccumulation in this matrix [7]. This makes it difficult for beekeepers to purchase uncontaminated foundation, and likely explains the persistence of contaminants in colonies even after comb replacement. Beeswax is also used by the cosmetic and pharmaceutical industries in numerous products like lipsticks, facial creams, pills coatings, chewing gum, and candles. Given that many of the pesticides detected in wax could pose endocrine disrupting effects, the development of methods to decontaminate wax will have a positive impact on human health as well. This study aims to improve managed honey bee colony health by developing methodology to decontaminate recycled wax and improve future work on wax decontamination.

Up to now, only few methods have been proposed to clean beeswax from pesticide residues. In this sense, methods developed propose the use of solid sorbents, like the patent US6586610B2 [8]. Serra Bonvehi and Jose Orantes-Bermejo [9], proved that activated charcoal is able to remove >95% of two organophosphorus —coumaphos and chlorfenvinphos—, widely detected in beeswax worldwide. However, this sorbent only removed fluvalinate pyrethroid a 35%. Our study demonstrated that organic solvent clean-up pose a wide scope, being able to eliminate pesticides belonging to many different families, and provides useful data of pesticide decontamination of beeswax by solvent extraction approach. Organophosphorus, but also carboxamide, pyrethroids and other pesticide families were removed from wax >95%. Pesticide content in the samples were reduced from μg·g ^−1^ levels to less than 10 ng·g^−1^ in all cases. Although beeswax texture is softer after solvent extractions, reconstituted into a useable form for cosmetic and pharmaceutical industries.

The procedure here proposed is a preliminary study on the possibilities of solvent extraction, and could be an effective alternative to remove pesticides from beeswax. As a pilot study, this method is feasible only on small scale because high amount of solvents are used during the extractions. A continuous solvent-solvent extractor design is needed to apply this methodology on a larger scale of wax production in order to minimize environmental harm and process cost. Hexane is easily evaporated, and its recovery during industrial processes would be of utmost importance to eliminate burdens to the environment. This would be the next mandatory step to fully implement this methodology within the beeswax sector, as an efficient, green and cheap method to get a proper clean-up of the beeswax from pesticide residues.
